# Characterization of the Micelle Formed by a Hydrophobically Modified Pullulan in Aqueous Solution: Size Exclusion Chromatography

**DOI:** 10.3390/polym13081237

**Published:** 2021-04-11

**Authors:** Jia Yang, Takahiro Sato

**Affiliations:** Department of Macromolecular Science, Osaka University, Toyonaka Osaka 560-0043, Japan; yangj17@chem.sci.osaka-u.ac.jp

**Keywords:** pullulan, size exclusion chromatography, multi-angle light scattering, micelle, flower necklace

## Abstract

Size exclusion chromatography equipped with a multi-angle, light-scattering online detector (SEC-MALS) measurements were carried out on a hydrophobically modified pullulan (PUL-OSA) with degrees of substitution (*DS*) of 0.14, 0.2, and 0.3 in 0.01 M aqueous NaCl to obtain the degree of polymerization (*N*_0_) dependence of the radius of gyration (〈*S*^2^〉^1/2^) for PUL-OSA in the aqueous NaCl. The result was consistent with the loose flower necklace model proposed in a previous study, and the increase in the chain size with introducing OSA groups was explained by the backbone stiffness of the loose flower necklace formed by PUL-OSA. For PUL-OSA samples with *DS* = 0.2 and 0.3, 〈*S*^2^〉^1/2^ obtained by SEC-MALS in a high *N*_0_ region deviated downward from 〈*S*^2^〉^1/2^ expected by the loose flower necklace model. This deviation came from a tiny amount of the aggregating component of PUL-OSA, taking a branched architecture composed of loose flower necklaces. Although the aggregating component of PUL-OSA was also detected by previous small angle X-ray scattering measurements, its conformation was revealed in this study by SEC-MALS.

## 1. Introduction

Size exclusion chromatography equipped with a multi-angle light scattering online detector (SEC-MALS) is one of the most powerful tools for the molecular characterization of polymer samples [1,2,3]. This method has also been applied to characterize associating and micelle-forming polymers in dilute solutions [4,5,6], although the number of reports on the applications of SEC-MALS to associating and micelle-forming polymers is much smaller than that on molecularly dispersed polymers in solution.

Associating polymers are usually polydisperse in size, and the merit of SEC-MALS is that it fractionates the polydisperse polymers into fractions with different sizes to characterize the associating components. The application of size exclusion chromatography (SEC) itself to micelle-forming polymer solutions began many years ago. In 1978, Booth et al. [7] studied a polystyrene-*b*-polyisoprene block copolymer in a selective solvent, *N*,*N*-dimethylacetamide. While the block copolymer was molecularly dispersed in the solvent at a high temperature, it formed a polymer micelle near room temperature. At intermediate temperatures, the SEC elution curve for the block copolymer became very broad, indicating that the micelle component of the block copolymer was partially dissociated into single chains in the SEC column. Because the polymer concentration is not uniform in the SEC column, it is rather difficult to analyze SEC data near the critical micellar temperature. A similar result was obtained by Špaček and Kubín [8], who studied a polystyrene-*b*-poly(ethylene-stat-butene)-*b*-polystyrene triblock copolymer in a dioxane-heptane mixture using SEC. Furthermore, Špaček [9] analyzed the elution curve of the triblock copolymer at intermediate temperatures to estimate the average time needed for the formation of micelles.

More recently, Hashidzume et al. [4] carried out a SEC-MALS experiment on amphiphilic random copolymer-bearing hydrophilic sulfonic acid and hydrophobic *n*-hexyl groups in an aqueous solution. This random copolymer solution contained a tiny amount of a large aggregating component, which was separated from the major micellar component by SEC to estimate the molar masses and amounts of not only the major micellar component but also the minor aggregating component. The peak of the SEC elution curve for the aggregating component was sharp, indicating that the aggregating component was stable against the dilution in the SEC column.

Yang and Sato [10,11] recently investigated a hydrophobically modified pullulan, octenyl succinic anhydride-modified pullulan (PUL-OSA), in aqueous solution by small-angle X-ray scattering (SAXS) and pyrene-probe fluorescence. The SAXS results indicated that the aqueous solutions of PUL-OSA contained the major micellar component, taking the (loose) flower necklace conformation and a small amount of a large aggregating component expressed by fractal aggregates. However, in batch measurements of light scattering, the scattering intensity from the aggregating component was so strong that we could not obtain information about the major micellar component from the batch measurements.

In the present study, we applied SEC-MALS to characterize the polymer micelle as well as the aggregating component formed by PUL-OSA in aqueous solution. The SEC-MALS method gives us the degree of polymerization dependence of the radius of gyration 〈*S*^2^〉^1/2^ characterizing the global conformation for the major micellar component and minor aggregating component of PUL-OSA. The separation of the major micellar and minor aggregating components by SEC makes it possible to estimate the 〈*S*^2^〉^1/2^ of both components by the MALS detector, which was difficult with batch measurements. While 〈*S*^2^〉^1/2^ data for the major micellar component have been compared with the loose flower necklace model proposed previously, 〈*S*^2^〉^1/2^ results for the minor aggregating component have provided us with information about the conformation of the aggregating component.

## 2. Materials and Methods

### 2.1. Materials

A polydisperse pullulan sample (PUL) and octenyl succinic anhydride (OSA) used in this study were purchased from TCI (Tokyo Chemical Industry Co., Ltd., Tokyo, Japan). Amphiphilic copolymer samples PUL-OSA with four different degrees of substitution (*DS*) were synthesized in the same procedure as previous reported [10,11] according to Eenschooten et al. [12,13,14,15,16]. That is, the PUL sample was firstly dissolved in Milli-Q water, and the pH of this PUL solution was adjusted to 8.5–9.0. OSA was added dropwise to this mild alkali solution of PUL under vigorous stirring, and the reaction solution was further stirred overnight at room temperature. By changing the molar ratio of OSA to glucose units of PUL in the reaction, PUL-OSA samples with the four different *DS* were obtained, as the Na salt form.

Each synthesized PUL-OSA sample was dissolved in D_2_O (1 mg/mL), and ^1^H-NMR was measured on the solution by a 400 MHz spectrometer of JEOL (JEOL Ltd., Tokyo, Japan) at 30 °C. Degrees of substitution *DS* for the four PUL-OSA samples were calculated from the peak area ratio of glucosidic proton signals to the octenyl terminal methylene proton signals [10,11]. Table 1 lists *DS* of the PUL-OSA samples determined by NMR.

### 2.2. SEC-MALS

SEC-MALS measurements were made for 0.01 M aqueous NaCl solutions of PUL and three PUL-OSA samples at a neutral pH, using a GPC-900 system of JASCO (JASCO Corporation, Tokyo, Japan) with columns SB-806M HQ and OHpak SB-G 6B of Shodex (Shodex Corporation, Tokyo, Japan), a DAWN HELEOS 2 (wavelength: 658 nm) MALS system of Wyatt (Wyatt Corporation, Santa Barbara, CA, USA), and an RI-930 refractive index (RI) detector. The flow rate of the eluent, 0.01 M aqueous solution of NaCl, was 0.5 mL/min, and the temperature was controlled at 40 °C.

The excess scattering intensity ∆*I_θ_* at the scattering angle *θ* and the excess refractive index ∆*n* of the solution over those of the solvent were measured as functions of the elution time. They were converted to the excess Rayleigh ratio *R_θ_* and the mass concentration *c* using the relations [5].

(1) $$R_{\theta }=\Phi_{\theta }\Delta I_{\theta },\;c={\left(\partial n/\partial c\right)}^{-1}\Delta n$$

where Φ*_θ_* is the instrument constant depending on *θ* and the refractive index of the eluent, and *∂n/∂c* is the refractive index increment of the polymer. The instrument constant Φ*_θ_* was determined using toluene as the standard material, and *∂n/∂c* was determined by differential refractometry. The results of *∂n/∂c* are listed in the last column of Table 1.

### 2.3. SAXS

SAXS measurements were conducted on solutions of PUL and four PUL-OSA samples in 0.05 M aqueous NaCl at the BL40B2 beamline of SPring-8 (JASRI, Hyogo, Japan). Polymer mass concentrations *c* were 5.51 × 10^−3^ g/cm^3^ (PUL), 9.08 × 10^−3^ g/cm^3^ (PDS14), 6.08 × 10^−3^ g/cm^3^ (PDS20), and 4.56 × 10^−3^ g/cm^3^ (PDS30). The wavelength of the X-ray, the camera length and the accumulation time were set to be 0.1 nm, 4 m and 180 s, respectively. A capillary made of quartz (2.0 mm, inner diameter) that contained test solutions was set in a heating block thermostated at 25 °C, and the intensity of the scattered X-rays was measured using a Dectris PILATUS2M instrument (DECTRIS, Baden, Switzerland) and circularly averaged. The SAXS excess Rayleigh ratio *R_θ,_*_X_ at the scattering angle *θ* and the optical constant *K*_e_ of SAXS were calculated in the same way as previously reported [10,11].

## 3. Results and Discussion

### 3.1. SEC-MALS

**Figure 1:**
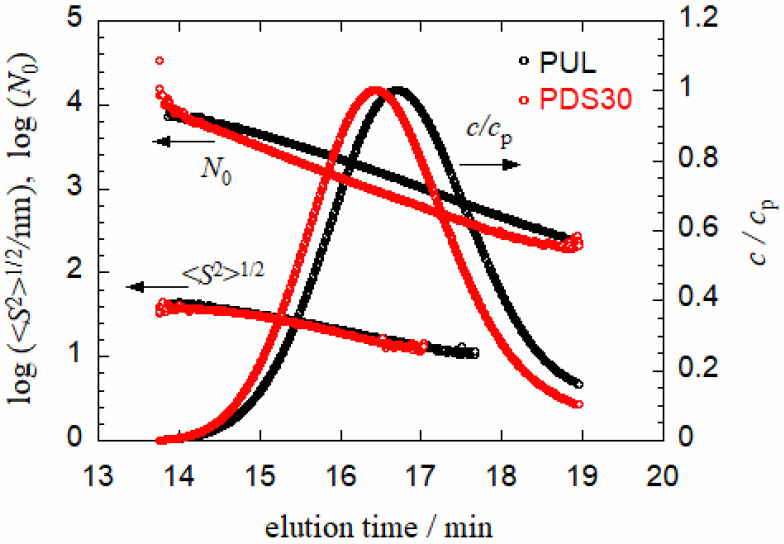
Elution curves (the polymer mass concentration *c* divided by the peak concentration *c*_p_) and elution time dependences of the degree of polymerization *N*_0_ and the radius of gyration 〈*S*^2^〉^1/2^ for a PUL-OSA sample (PDS30) and PUL in 0.01 M aqueous NaCl.

Figure 1 compares SEC elution curves and elution time dependences of the degree of polymerization *N*_0_ and of the radius of gyration 〈*S*^2^〉^1/2^ between a PUL-OSA sample, PDS30, (red circles) and PUL (black circles) in 0.01 M aqueous NaCl at 25 °C. Both elution curves are single-peaked, and the elution curve for the PUL-OSA sample slightly shifts to the shorter elution time side than that for PUL. While the *N*_0_ line (except for shortest and longest elution time regions) for the PUL-OSA sample also slightly shifts to the shorter elution time side, the 〈*S*^2^〉^1/2^ lines almost overlap for both samples, demonstrating that the elution time is determined by the polymer chain dimension.

**Figure 2:**
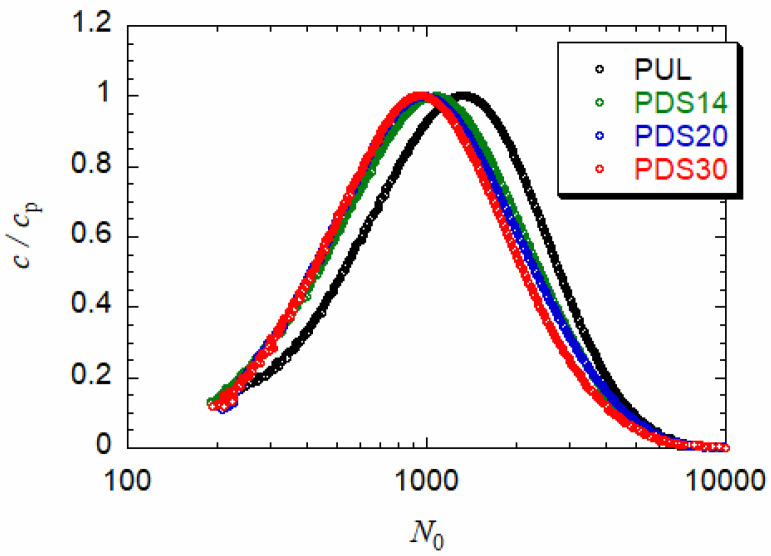
Distributions of the degree of polymerization *N*_0_ of PUL and PUL-OSA samples.

Figure 2 shows distributions of *N*_0_ for three PUL-OSA samples with different *DS* and the PUL sample before the substitution reaction. The conversion of the abscissa from the elution time to *N*_0_ was made by using the *N*_0_-elution time relations given in Figure 1. (Because of low scattering intensities, we do not show data at low *N*_0_ (<200) or long elution times (>19 min)). All distribution functions are single-peaked, but those for PUL-OSA shift slightly to the lower *N*_0_ side with increasing *DS*. Weight average degrees of polymerization *N*_0,w_ and dispersity index *Ð* (the ratio of the weight to the number of average degrees of polymerization) calculated from the distribution functions in Figure 2 are listed in Table 2. Although the substitution reaction of PUL-OSA was made under the mild alkali condition, and also as demonstrated in a previous study [11], *N*_0,w_ of narrow distribution PUL-OSA samples determined by SAXS did not decrease with *DS*, while *N*_0,w_ listed in Table 2 slightly decreased with increasing *DS*. This decrease in *N*_0,w_ may come from the adsorption of small amounts of a higher degree of polymerization fractions or aggregates [10,11] of PUL-OSA samples in the SEC column. In fact, the total concentration of PUL-OSA eluted from SEC slightly decreased with increasing *DS*.

**Figure 3:**
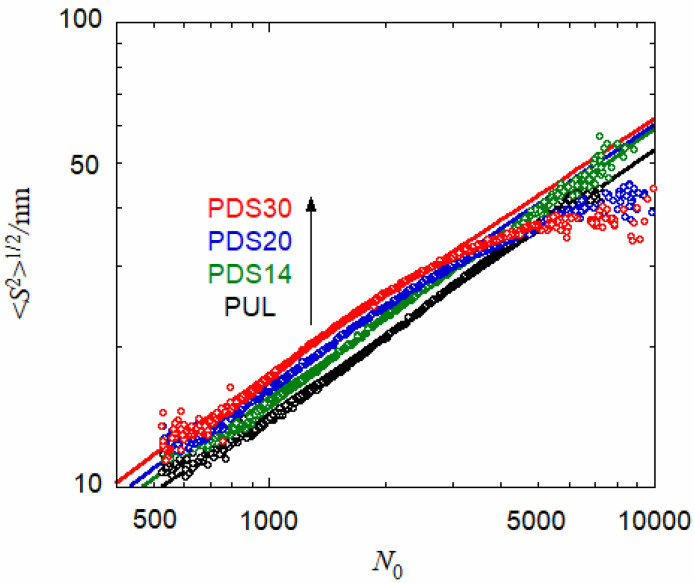
Degree of polymerization dependence of the radius of gyration 〈*S*^2^〉^1/2^ for PUL and PUL-OSA with three different *DS* in 0.01 M aqueous NaCl. Solid curves, calculated by Equation (11) with *q* = 1.4 nm (PUL), 1.57 (PDS14), 1.52 (PDS20), and 1.53 (PDS30) along with other parameters listed in Table 3.

Figure 3 plots 〈*S*^2^〉^1/2^ vs. *N*_0_ for PUL and PUL-OSA with three different *DS*, obtained by SEC-MALS (cf. Figure 1). (It is noted that the relation of 〈*S*^2^〉^1/2^ vs. *N*_0_ is not essentially affected by the weak adsorption of polymer in the SEC column.) With increasing *DS*, data points go up at *N*_0_ < 2500, indicating that the PUL-OSA chain takes slightly more expanded conformation than PUL in aqueous medium. The slight chain expansion of PUL-OSA by introducing hydrophobic OSA groups was also demonstrated by SAXS in a previous study [10] but forms a striking contrast with an amphiphilic alternating copolymer with vinyl chain backbones, of which conformations shrink remarkably by introducing hydrophobic monomer units [17]. For PUL-OSA samples PDS20 and PDS30 with higher *DS*, data points at high *N*_0_ deviate from the power-low dependence, indicated by straight lines, and 〈*S*^2^〉^1/2^ at *N*_0_ > 5000 are smaller than that for PUL.

### 3.2. SAXS

**Figure 4:**
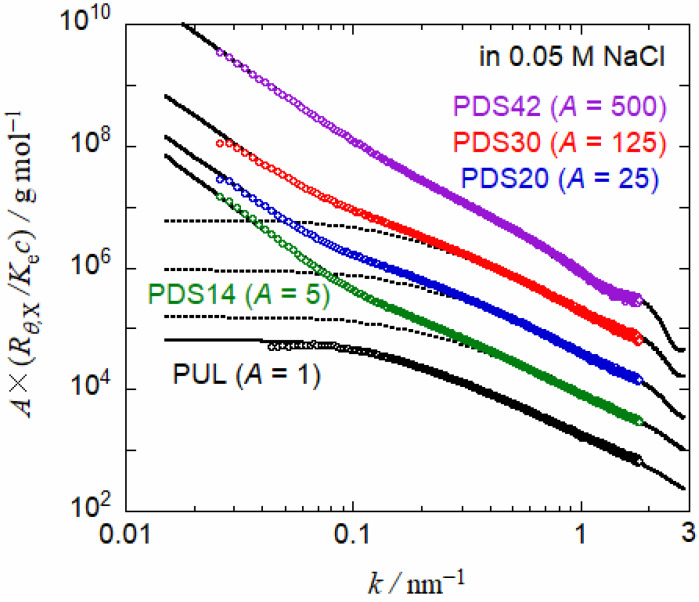
SAXS profiles in 0.05 M aqueous NaCl of PUL and PUL-OSA samples with four different *DS*. Profiles shifted vertically other than PUL with the shift factor *A* = 1, 5, 25, 100, 500. Solid curves indicate theoretical values for the loose flower necklace model (see text). The dotted curve indicates theoretical values of theoretical scattering function without aggregating component.

SAXS profiles for PUL and four PUL-OSA samples in 0.05 M aqueous NaCl are shown in Figure 4. PUL and three PUL-OSA samples are the same used for the above SEC-MALS experiments. Here, *R_θ_*_X_ is the excess Rayleigh ratio of SAXS, *K*_e_ is the optical constant calculated from the partial specific volume and the excess electron density of PUL-OSA depending on *DS* [10], *k* is the magnitude of the scattering vector, and *c* is the polymer mass concentration. The profile for PUL has a plateau in a low *k* region and monotonically decreases with increasing *k*, characteristic to flexible polymer chains. On the other hand, the SAXS profile for PDS42 is a decreasing function of *k* and shows a shallow minimum around *k* ~ 1.3 nm^−1^. This minimum comes from the scattering from the hydrophobic core of octenyl groups with low electron density. As shown in previous studies [10,11], the minimum is more pronounced with increasing *DS*. The upswings for PUL-OSA profiles in the low *k* region indicate a small amount of large aggregates, as observed in previously studies [10,11].

The SAXS profiles for PDS14, PDS20, and PDS30 resemble that for PUL in a high *k* region, and that for PDS42 in a low *k* region. Similar SAXS profiles were previously reported for narrow-distribution PUL-OSA samples with similar *DS* [11]. The previous paper demonstrated that SAXS profiles for PUL-OSA with low *DS* can be fitted to the loose flower necklace model explained below.

### 3.3. Fitting of the SAXS Profiles by the Loose Flower Necklace Model

**Figure 5:**
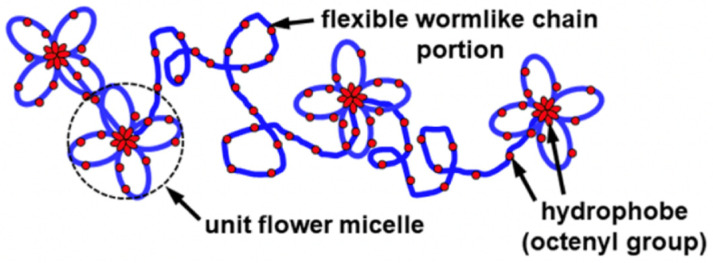
Schematic illustrations of the loose flower necklace model.

As an intermediate model between the flower necklace and wormlike chain models, the previous study introduced the loose flower necklace model [11], schematically illustrated in Figure 5. In this loose flower necklace model, the polymer chain is divided into *n*_c_ + *n_l_* sub-chains, and *n*_c_ and *n_l_* sub-chains form unit flower micelles and take the wormlike chain conformation, respectively. The degree of polymerization of each sub-chain is denoted as *N*_ou_, which is the optimum number of monomer units (glucose residues) forming the unit flower micelle. (The optimum number corresponds to the aggregation number for spherical micelles formed by low molar mass surfactants [18].) Thus, the degree of polymerization *N*_0_ of the total chain is related to *n*_c_ + *n_l_* by

(2) $$N_{0}/N_{0u}=n_{c}+n_{l}$$

The unit flower micelle is characterized in terms of the mean radius of the hydrophobic core ${\bar{R}}_{\mathrm{core}}$ and the height of the minimum loop chain *d*_loop_. (The size distribution of the hydrophobic core is characterized by ${\bar{R}}_{\mathrm{core}}$ and the variance *σ*^2^ [10,11].) Using the wormlike chain statistics, *d*_loop_ is related to the persistence length *q* of the polymer chain by

(3) $$d_{\mathrm{loop}}=0.62q$$

and the mean radius of the micelle $\bar{R}$ is calculated by

(4) $$\bar{R}={\bar{R}}_{\mathrm{core}}+d_{\mathrm{loop}}$$

The full flower necklace with *N*_0_*/N*_0u_ = *n*_c_ is regarded as a wormlike chain with the persistence length *q*_FN_ and the contour length *L*_FN_. The latter quantity is calculated by:

(5) $$L_{\mathrm{FN}}=2n_{c}\bar{R}$$

On the other hand, the full wormlike chain with *N*_0_*/N*_0u_ = *n_l_* is characterized by the persistence length *q*, the contour length *L* of the total chain, the excluded volume strength *B*, and the chain thickness *d*_b_. The contour length *L* is calculated from the contour length per monomer unit *h* (for PUL, *h* = 0.35 nm [19]) by:

(6) $$L=N_{0}h$$

The scattering function for the loose flower necklace consisting of *n*_c_ unit flower micelles and *n_l_* wormlike chain portions may be approximately written by the following interpolation formula [11]

(7) $$P(k)=\frac{1}{{\left(n_{c}+n_{l}\right)}^{2}}{\left[n_{c}P_{\mathrm{FN}}{}^{1/2}(k)+n_{l}P_{\mathrm{WC}}{}^{1/2}(k)\right]}^{2}$$

where *P*_FN_(*k*) is the scattering function for the full flower necklace (*n_l_* = 0), and *P*_WC_(*k*) is the scattering function for the full wormlike chain (*n*_c_ = 0). The scattering functions *P*_FN_(*k*) and *P*_WC_(*k*) are given in a previous paper [11]. The function *P*_FN_(*k*) contains the relative difference in the contrast factor ∆*ρ*_c._

(8) $$\Delta \rho_{c}\equiv \frac{\Delta \rho_{\mathrm{core}}-\Delta \rho_{\mathrm{shell}}}{\Delta \rho_{\mathrm{shell}}}$$

Here, ∆*ρ*_core_ is the excess electron densities at the core region, and ∆*ρ*_shell_ is the excess electron densities at the loop (shell) region, respectively. We assume that hydrophobes outside the hydrophobic core belong to the shell region.

At a finite polymer concentration, the scattering function is affected by the inter-molecular interference. Moreover, as shown in Figure 4, aqueous PUL-OSA solutions contain a small amount of a large aggregating component. Thus, the scattering function of aqueous PUL-OSA may be written in the form [10,11,20]

(9) $$\frac{R_{\theta ,X}}{K_{e}c}=\frac{\left(1-w_{\mathrm{agg}}\right)M_{0}N_{0}P(k)}{1+2A_{2}M_{0}N_{0}P(k)c}+w_{\mathrm{agg}}M_{w,\mathrm{agg}}P_{z,\mathrm{agg}}(k)$$

where *M*_0_ is the average molar mass per glucose residue of PUL-OSA (cf. Table 1), *N*_0_ is the degree of polymerization of the PUL-OSA chain, *A*_2_ is the second virial coefficient, and *w*_agg_, *M*_w,agg_, and *P*_z,agg_(*k*) are the weight fraction, weight average molar mass, and z-average particle scattering function of the aggregating component, respectively. According to a previous study [10,11], we assume that the aggregating component is fractal-like, of which scattering function is given by [21]

(10) $$w_{\mathrm{agg}}M_{w,\mathrm{agg}}P_{z,\mathrm{agg}}(k)=K_{\mathrm{fractal}}k^{-\alpha }$$

with a constant *K*_fractal_ proportional to *w*_agg_ *M*_w,agg_ and the fractal exponent *α*. Here, *w*_agg_ and *M*_w,agg_ are not separable. Although this Equation (10) only holds in a limited (low and high) *k* region, it is assumed that the low *k* limit is out of the experimental *k* region, and the high *k* limit is hidden by the scattering contribution of the major component.

Referring previous fitting results of SAXS scattering functions of PUL-OSA and PUL [10,11], we have chosen parameters of the flower micelle and wormlike chain portions of PUL-OSA as well as wormlike chain parameters of PUL, listed in Table 3a. Although weight average degrees of polymerization *N*_0,w_ were determined by SEC-MALS for three PUL-OSA samples (cf. Table 2), they were affected by the adsorption of the polymer in the SEC column as mentioned above. Thus, we have chosen *N*_0_ for all PUL-OSA samples to be equal to *N*_0,w_ (= 1510) for PUL. By use of remaining parameters, including those in Equation (9) for each PUL-OSA sample listed in Table 3b, we obtain solid curves in Figure 4, which satisfactorily agree with experimental SAXS profiles for all PUL-OSA and PUL samples. Dotted curves in Figure 4 for three PUL-OSA samples indicate theoretical values calculated by Equation (9) with *w*_agg_ = 0. It turns out that the sharp increases of the scattering functions for PUL-OSA at *k* < 0.2 nm^−1^ arise from the small amount of the large aggregating component.

### 3.4. Comparison of the SEC-MALS Results with the Loose Flower Necklace Model

By expanding the scattering function *P*(*k*) in Equation (7) in power series of *k*^2^ [22], we have the following radius of gyration for the loose flower necklace:

(11) $${\langle S^{2}\rangle }_{\mathrm{LFN}}=\frac{n_{c}\left({\langle S^{2}\rangle }_{\mathrm{KP}}+{\langle S^{2}\rangle }_{\mathrm{flower}}\right)+n_{l}\left({\langle S^{2}\rangle }_{\mathrm{PS}}+\frac{3}{20}d_{b}{}^{2}\right)}{n_{c}+n_{l}}$$

where 〈*S*^2^〉_KP_ and 〈*S*^2^〉_PS_ are mean square radii of gyration of the unperturbed wormlike chain for the full flower necklace and of the perturbed wormlike chain for the loose flower necklace at *n*_c_ = 0, respectively, and 〈*S*^2^〉_flower_ is the mean radius of gyration of the unit flower micelle. The mean square radius of gyration 〈*S*^2^〉_KP_ can be calculated from *q*_FN_ and *L*_FN_ given by Equations (4) and (5) with ${\bar{R}}_{\mathrm{core}}$, *d*_loop_, and *n*_c_ listed in Table 3, while 〈*S*^2^〉_PS_ can be calculated from *h*, *q*, and *B* in Table 3a, as functions of *N*_0_ (cf. Equation (6) to calculate the contour length of the wormlike chain *L*) [21]. From Equation (8) in [11], the mean square radius of gyration 〈*S*^2^〉_flower_ of the unit flower micelle is calculated by

(12) $${\langle S^{2}\rangle }_{\mathrm{flower}}\equiv \frac{3}{5\sqrt{\pi }}\int \frac{R_{\mathrm{core}}{}^{5}\Delta \rho_{c}+R^{5}}{R_{\mathrm{core}}{}^{3}\Delta \rho_{c}+R^{3}}\exp\left(-x^{2}\right)dx,\;x\equiv \frac{R_{\mathrm{core}}-{\bar{R}}_{\mathrm{core}}}{\sqrt{2}\sigma }$$

with ${\bar{R}}_{\mathrm{core}}$, *d*_loop_, *σ*, and ∆*ρ*_c_ listed in Table 3a.

When parameter values listed in Table 3 are used to calculate 〈*S*^2^〉_LFN_ as a function of *N*_0_, the theoretical results slightly overestimate the experimental 〈*S*^2^〉^1/2^ for PUL-OSA and PUL (at *N*_0_ < 2000 for PDS20 and PDS30 and at all *N*_0_ for PUL and PDS14) shown in Figure 3. To obtain the best fit, *q* of the wormlike chain portion should be slightly decreased from those given in Table 3a. Four solid lines in Figure 3 show theoretical values calculated by Equation (11) with *q* = 1.4 nm (PUL), 1.57 (PUL (PDS14), 1.52 (PDS20), and 1.53 (PDS30). (If these *q* values were used to calculate SAXS scattering functions, the theoretical values slightly overestimated the experimental results shown in Figure 4 at high *k*.) The difference in *q* estimated by SEC-MALS and SAXS does not come from the difference in the NaCl concentration between the two experiments, because the lower NaCl concentration should increase *q*.

We did not consider the heterogeneity in the distribution of octenyl groups along the PUL chain in the above discussion. As mentioned in a previous paper [11], the heterogeneity becomes important at low *DS*, especially at *DS* < 0.12 [11], but our PUL-OSA samples have average *DS* higher than this critical *DS*.

**Figure 6:**
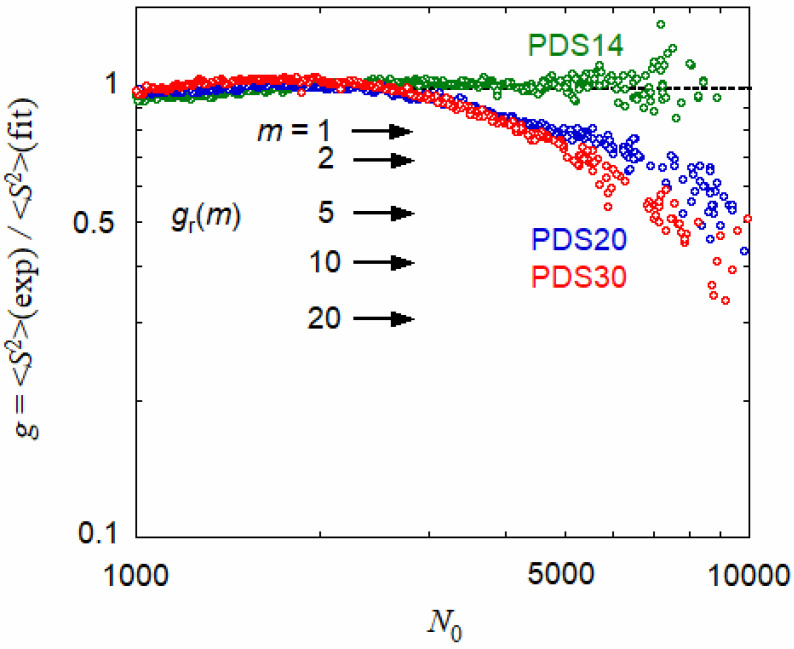
Ratio *g* of the experimental mean square radius of gyration for three PUL-OSA samples to the theoretical calculated by Equation (11) with the parameters mentioned in the caption of Figure 3 (cf. solid curves in Figure 3). Arrows indicate theoretical *g*-factors for the branched polymer of the random-type, calculated by Equation (13) for *f* = 4 and different *m*.

In Figure 3, data points for PDS20 and PDS30 at *N*_0_ > 2000 deviate from the solid curves. Figure 6 shows ratios *g* of the experimental mean square radius of gyration for PUL-OSA samples to the theoretical calculated by Equation (11), with the parameters mentioned in the caption of Figure 3. Although experimental errors are rather large at high *N*_0_, the ratios for PDS14 are almost unity over the entire *N*_0_ range, and those for PDS20 and PDS30 decrease from unity with increasing *N*_0_. The decrease is more pronounced for PDS30 than PDS20 at higher *N*_0_.

The smaller experimental 〈*S*^2^〉 than the theoretical for PDS20 and PDS30 at high *N*_0_ indicates the branching of the loose flower necklace. The SEC may fractionate the aggregating component, inducing the sharp increases in the SAXS scattering functions in low *k* region shown in Figure 4, and the aggregating component may be eluted in a shorter elution time region, corresponding to the high *N*_0_ region in Figure 4 or Figure 6. Thus, the result in Figure 6 implies that the aggregating components of PDS20 and PDS30 are the branched loose flower necklace.

In Figure 4, SAXS profiles in the low *k* region were fitted by Equation (10) for fractal aggregates. However, this equation is a just phenomenological equation, not specifying any concrete structure of the aggregate. Furthermore, the weight fraction *w*_agg_ of the aggregating component of PUL-OSA is so small (cf. Table 3b) that the characterization of the aggregating component is not easy. The suggestion of the branched loose flower necklace for the aggregating component of PUL-OSA with higher *DS* by SEC-MALS has not been supported by other experimental evidence at present.

Kurata and Fukatsu [23] calculated the *g*-factor for the branched polymer of the random-type branching with *m* branch points of the functionality *f* and *p* Gaussian sub-chains of random distribution of the degree of polymerization. Their result is given by

(13) $$\begin{array}{c} g_{r}(m)=\frac{6}{p\left(p+1\right)\left(p+2\right)}\left[p^{2}+h\left(f,m\right)\right]\\ p=\left(f-1\right)m+1\\ h\left(f,m\right)\equiv \frac{1}{2}{\left(f-1\right)}^{2}m\left(m-1\right)\sum_{\nu =1}^{m-1}\frac{\left(fm-m-\nu \right)!\left(m-2\right)!}{\left(fm-m\right)!\left(m-\nu -1\right)!}{\left(f-1\right)}^{\nu -1}\left(f\nu -2\nu +f\right)\nu \end{array}$$

Arrows in Figure 6 indicate the theoretical *g*-factors *g*_r_(*m*) for the random-type branching, calculated by Equation (13) with *f* = 4 and different *m* = 1–20. When three (six) PUL-OSA chains with degree of polymerization = 1510 form a trimer (hexamer), *N*_0_ is equal to 4530 (9060), where the experimental *g* values for PDS20 and PDS30 in Figure 6 are close to *g*_r_(*m*) at *m* = 2 (5) indicated by the arrow. It is reasonable that the number of branch points with *f* = 4 is equal to the aggregation number minus unity. (For example, a randomly aggregating dimer has a single branch point.) 

## 4. Conclusions

SEC-MALS measurements were carried out on a hydrophobically modified pullulan PUL-OSA with *DS* = 0.14, 0.2, and 0.3 in 0.01 M aqueous NaCl. Although a small fraction of PUL-OSA was adsorbed in the SEC column, the main component of PUL-OSA is eluted according to the size of the PUL-OSA chain. The degree of polymerization dependence of the radius of gyration 〈*S*^2^〉^1/2^ for PUL-OSA (except for PDS20 and PDS30 at high *N*_0_) obtained by SEC-MALS can be fitted by the loose flower necklace model (cf. Figure 6) almost consistently with SAXS scattering functions of the same PUL-OSA samples. The chain size increases slightly by introducing OSA groups, mainly due to the stiffness of the flower necklace backbone.

Radii of gyration 〈*S*^2^〉^1/2^ for PDS20 and PDS30 obtained by SEC-MALS deviate downward from the loose flower necklace model at high *N*_0_, which may come from the aggregating component of PUL-OSA taking a branched architecture of the loose flower necklace. SEC-MALS provides us this important information, which cannot be obtained by other experimental techniques, including SAXS.

## Figures and Tables

**Table 1:** Degrees of substitution (*DS*), the average molar masses per unit glucose residue (*M*_0_), and the refractive index increments (*∂n/∂c*) of polydisperse pullulan (PUL) and octenyl succinic anhydride-modified pullulan (PUL-OSA) samples used in this study.

Sample	*DS*	*M*_0_	*∂n/∂c*/g^−1 ^cm^3^
PUL	0	162	0.135
PDS14	0.14	194	0.137
PDS20	0.20	208	0.138
PDS30	0.30	232	0.140
PDS42	0.42	259	-

**Table 2:** Molecular characteristics of PUL and PUL-OSA samples.

Sample	*DS*	*N*_0,w_	*Ð*
PUL	0	1510	1.63
PDS14	0.14	1270	1.69
PDS20	0.20	1200	1.69
PDS30	0.30	1120	1.63

**Table:** (a)

Polymer	Flower Micelle Portion				Wormlike Chain Portion			
	${\bar{R}}_{\mathrm{core}}/\mathrm{nm}$	*d*_loop_/nm ^a^	*σ*	∆*ρ*_c_	*h*/nm	*q*/nm	*B*/nm	*d*_b_/nm
PUL	-	-	-	-	0.35	1.5	0.3	1.0
PUL-OSA	1.4	1.12	0.25	−1.66	0.35	1.8	0.5	1.0

**Table:** (b)

Sample	*N*_0_	*N*_0u_	*n*_c_	*n_l_*	*q*_FN_/nm	*A*_2_^b^	*w*_agg_	*K*_fractal_	*α*
PDS14	1510	70	1.3	20	1.8	1.6	0.01	1 × 10^4^	−2.82
PDS20		40	15	23		2.0	0.008		−2.66
PDS30		30	25	25		2.0	0.0085		−2.63

^a^ Calculated by Equation (3) with *q* = 1.8 nm; ^b^ In units of 10^−3^ cm^3^ g^−2^mol.

## Data Availability

The data presented in this study are available on request from the corresponding author.
